# Confidence modulates exploration and exploitation in value-based learning

**DOI:** 10.1093/nc/niz004

**Published:** 2019-05-08

**Authors:** Annika Boldt, Charles Blundell, Benedetto De Martino

**Affiliations:** 1 Institute of Cognitive Neuroscience, University College London, 17 Queen Square, London, UK; 2 Department of Psychology, University of Cambridge, Downing Street, Cambridge, UK; 3 Google DeepMind, London, UK

**Keywords:** uncertainty, confidence, metacognition, exploration–exploitation dilemma, value-based choice

## Abstract

Uncertainty is ubiquitous in cognitive processing. In this study, we aim to investigate the ability agents possess to track and report the noise inherent in their mental operations, often in the form of confidence judgments. Here, we argue that humans can use uncertainty inherent in their representations of value beliefs to arbitrate between exploration and exploitation. Such uncertainty is reflected in explicit confidence judgments. Using a novel variant of a multi-armed bandit paradigm, we studied how beliefs were formed and how uncertainty in the encoding of these value beliefs (belief confidence) evolved over time. We found that people used uncertainty to arbitrate between exploration and exploitation, reflected in a higher tendency toward exploration when their confidence in their value representations was low. We furthermore found that value uncertainty can be linked to frameworks of metacognition in decision making in two ways. First, belief confidence drives decision confidence, i.e. people’s evaluation of their own choices. Second, individuals with higher metacognitive insight into their choices were also better at tracing the uncertainty in their environment. Together, these findings argue that such uncertainty representations play a key role in the context of cognitive control.

**Highlights**

- Humans use uncertainty to arbitrate between exploration and exploitation.
- Individuals with higher metacognitive abilities are better at tracing the uncertainty.
- Belief confidence in both the chosen and unchosen item impact on decision confidence.

## Introduction

All cognitive computations are plagued by uncertainty (Bach and Dolan 2012). On the one hand, uncertainty can be inflicted on our cognitive systems through external sources, e.g. when we perceive noisy information in the environment. On the other hand, uncertainty can arise directly from the way the brain processes information. Recent research has supported the view of a signal-inherent representation of uncertainty, suggesting a coding scheme in which the reliability of a signal is represented together with its average strength (e.g. Ma *et al.* 2006; Ma and Jazayeri 2014; Van Bergen *et al.* 2015). Such a coding scheme allows the decision maker to more efficiently integrate new evidence by giving more weight to a reliable evidence source and discounting information from a source identified as unreliable. Such flexible weighting of evidence might take place automatically and without agents being aware of it. A wealth of empirical work has found that humans can accurately trace uncertainty and report the level of confidence in their judgments (Peirce and Jastrow 1884; Henmon 1911; Audley 1960; Shea *et al.* 2014; Meyniel *et al.* 2015b; Guggenmos *et al.* 2016). However, different sources of evidence are by no means to be considered equal. A recent study by Boldt *et al.* (2017) found that evidence reliability, operationalized as the variability of colors in a stimulus array, affected confidence more than evidence strength, operationalized as the distance of the average color from a decision boundary.

Classically, confidence has been investigated focusing on only the last stage of the decision-making process, with confidence reflecting the internal belief as to whether the chosen option was the “good” or “correct” one (e.g. Pleskac and Busemeyer 2010; Baranski and Petrusic 1994; Fleming *et al.* 2010). At more conceptual level, Pouget *et al.* (2016) proposed that we should make a distinction between decision confidence that tracks the probability of an action to be correct, and certainty that indexes the degree of uncertainty in a representation. Following this theoretical proposal in this study, we experimentally measure how these two quantities separately evolve during learning. We define “decision confidence” as the confidence that an action (e.g. choosing the most valuable bandit) is correct. We define “belief confidence” as the agent’s internal readout of the uncertainty inherent to her belief about the value of each bandit, related to the concept of “certainty” proposed in the framework by Pouget *et al.* (2016). More specifically, when people assign a value to an object on which they base their preferences and choices, those beliefs can vary with regard to how precise they are: on our first day at work, we might guess that we will like our new job, but several months later our certainty in that belief might have increased, resulting in a highly precise value belief representation. The study of decision confidence reaches back to the early beginnings of experimental psychology (e.g. Peirce and Jastrow 1884; Fullerton and Cattell 1892; Griffing 1895; Henmon 1911). Much of this work had focused perception and memory (Koriat *et al.* 1980; Nelson and Narens 1990; Gigerenzer *et al.* 1991; Baranski and Petrusic 1995; Hertzog and Hultsch 2000; Pleskac and Busemeyer 2010). At the theoretical level, one of the most successful framework was proposed by Douglas Vickers that defined confidence as the “balance of evidence”, the difference between two dynamic accumulators at the time of the choice (the so-called “Vickers race model”; Vickers and Packer 1982). Recently, there has been a renaissance of these seminal earlier lines of investigation and the study of confidence has been moved beyond the context of perception and memory to include (as in the present study) learning and value-based choice (e.g. De Martino *et al.* 2013; Koriat 2013; Lebreton *et al.* 2015; Meyniel *et al.* 2015a). Similarly, confidence in attitudes and value beliefs has been investigated both with behavioral (e.g. Brim 1955; Smith and Swinyard 1988) and recent neuroimaging studies (Lebreton *et al.* 2015; De Martino *et al.* 2017). More recently, it has been shown that it is possible to disentangle the contribution of certainty and confidence on perceptual choice at the neurocomputational level (Bang and Fleming 2018). However, how these two quantities interplay in the context of value-based learning has not been studied. This is of particular importance in this context of value-based decision making, where choices are based on internally constructed beliefs about the value of two or more alternatives. Does confidence in the value beliefs we hold have an effect on our choices? And does such belief confidence feed into our decision confidence?

Using a novel variant of a multi-armed bandit task, we show that we can reliably measure how confidence in people’s beliefs evolves over the course of learning. The aim of our study was 2-fold. First, we investigate the links between people’s belief confidence and decision confidence. The second goal of our study was to investigate how an agent uses the explicit representations of the uncertainty inherent in value representations (belief confidence) to arbitrate between different behavioral strategies. Previous research has highlighted the role of confidence signals as internal teaching signals that support cognitive control (Fernandez-Duque *et al.* 2000), learning (Guggenmos *et al.* 2016), and social interactions (Bahrami *et al.* 2010). Here, we are focusing on higher-order action selection, the so-called “exploration–exploitation trade-off” (Schumpeter 1934; Sutton and Barto 1998; Cohen *et al.* 2007; Kolling *et al.* 2012). Arbitrating optimally between exploration and exploitation is not trivial and different algorithms for achieving a good balance between these extreme behavioral strategies have been discussed in the machine learning literature. A principled and efficient way of arbitrating between these modes is to deploy exploration toward the options or actions regarding which the agent is more uncertain (Gittins and Jones 1974; Dayan and Sejnowski 1996). Experimental work has shown that people can implement such sophisticated strategies, which take into account the level of uncertainty in their environment (Daw *et al.* 2006; Frank *et al.* 2009), even if there are substantial inter-individual differences (Badre *et al.* 2012).

Here, we present the results from two studies in which participants continuously faced two lotteries (two-armed bandits), each associated with a different average outcome. The participants’ task was to maximize their earnings by choosing the most rewarding bandit. Furthermore, participants had to rate the value of the lotteries together with the confidence they held in this value belief (belief confidence), which we predicted would guide their choices. From time to time, participants were furthermore asked to freely choose between the two lotteries and to rate how accurate they regarded their choice (decision confidence). The first experiment focused on the development of preferences and confidence judgments over time. The second study focused on the decision stage and the influence of belief confidence on the trade-off between exploration and exploitation. Taken together, our findings argue that the explicit representation of uncertainty expressed through confidence report plays a key role in arbitrating between complex decision strategies.

## Materials and Methods

### Participants

In Experiment 1, we tested 22 participants, 20 of which were female, and 3 were left-handed. Participants’ ages ranged from 18 to 32 years (*M *=* *24.0). One participant had to be excluded because during debriefing she reported that she had tracked the value of the bandits with a piece of paper. The final sample therefore included 21 participants. For Experiment 2, we tested 30 participants, 15 of whom were female, and 1 was left-handed. The participants’ ages from Experiment 2 ranged from 19 to 34 (*M *=* *26.4). No participants were excluded in this experiment.

For both experiments, all participants reported normal or corrected-to-normal vision, were English-proficient, and reported that they had no psychiatric or neurological disorders or gambling addiction. All participants gave informed consent and were reimbursed for their time (£10/h). Each session lasted approximately 90 min, including instruction and debriefing. In addition, participants also received performance-dependent rewards (Experiment 1: *M* = £8.82; min = £8.44; max = £8.97; Experiment 2: *M* = £8.71; min = £8.17; max = £9.11). All procedures were approved by the local ethics committee.

### Task and procedures

**Figure 1.**
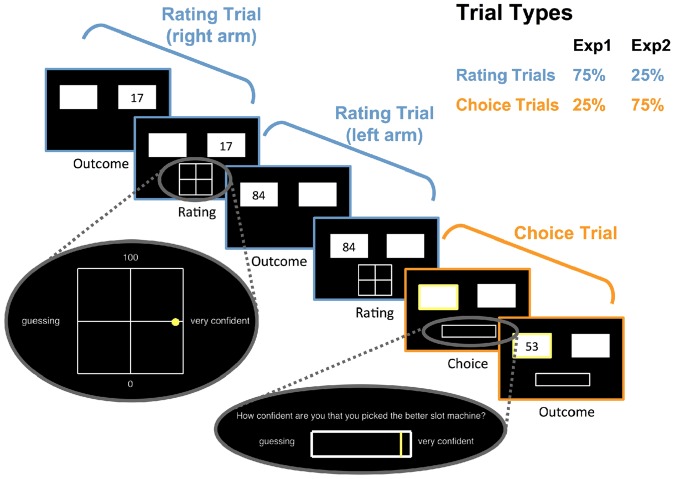
Schematic representation of the task structure, showing a typical sequence of trials: people were faced with both rating (blue) and choice (orange) trials. During rating trials, they observed outcomes randomly from one arm of the two-armed bandit, represented as squares. Participants then rated the average value of this arm and their confidence in this value-belief estimate on a 2D grid. During choice trials, participants freely chose one arm of the bandit, rated their confidence in this decision and were then shown the reward. In Experiment 1, 75% of trials were rating trials and 25% trials were choice trials, with both trial types intermixed randomly. In Experiment 2, these proportions were reversed.

Experiment 1 comprised 75% rating trials and 25% choice trials. During rating trials, participants were first presented with the outcome of one of the randomly chosen arms of the bandit. They then had to rate the value belief associated with this arm (average number of points that could be won from this machine) and their confidence in this estimate. We used a 2D grid scale to collect these measurements. Value-belief judgments ranged from 0 to 100 points, belief confidence judgments from “guessing” to “very confident”. Participants were told that during rating trials, they would observe another person gamble at one of the two slot machines. We chose to use this cover story to make the task more engaging for the participants (increasing their motivation) and to stress that the two arms of the bandits were independent by comparing them to actual separated slot machines. Unbeknownst to the participant, this person chose one of the slot machines randomly with equal probability. During choice trials, participants could freely choose one of the two arms of the bandit and were then asked to rate their confidence in being correct in their choice before seeing the outcome of the trial. Decision confidence ratings were given on a scale that ranged from “guessing” to “very confident”. It should be noted that with such a scale, participants are unable to report any mistakes they detected in their own decisions. However, we decided to use this scale since it was more intuitive and we reasoned that such mistakes were likely to be rare in this experiment (participants gained a considerable amount of information from rating trials and the task was not speeded). Furthermore, we reasoned that the confidence for most choices would likely be “guessing” or higher, and we wanted to maximize resolution with which decision confidence was measured. Moreover, we wanted to match the decision confidence scale to the belief-confidence scale. Points won on those trials contributed toward the final reimbursement sum participants received after the experiment and they were instructed to carefully use the information previously gained when making their choices. Both trial types were intermixed randomly. Figure 1 shows an example of three trials (two rating and one choice trial).

In total, participants completed 600 trials, grouped into blocks of different lengths (20–60 trials long). Participants were thus unable to predict how many evidence samples they would be able to collect in the present block, allowing us to avoid the possibility that they would adjust their risk-taking behavior given how close they were to the end of the block (Kolling *et al.* 2014). For each block, beta distributions were taken to generate the rewards of the two arms of the bandit. Beta distributions can be parametrized by two positive shape parameters, α and β, which were set to [(α = 1; β = 3), (α = 2; β = 3), (α = 3; β = 3), (α = 3; β = 2), (α = 3; β = 1)] in the present study. Out of these five possible distributions, two were taken to generate the rewards in each block. These parameter ranges were chosen so that the resulting distributions had different skews away from uniform while being unimodal. The resulting samples were multiplied by 100 to achieve rewards bounded between 0 and 100 points. Prior to the experiment, participants completed three practice blocks, introducing them to choice and rating trials separately (5 trials each) and together (20 trials). Importantly, all participants completed exactly the same blocks, shuffled with regard to the order in which they appeared. The gamble outcomes that participants were presented with were thus the same for everyone, only dependent on their choices.

A key question we aimed to address with Experiment 2 was whether people would use their belief confidence to arbitrate between exploration and exploitation. While the high number of rating trials (75%) in Experiment 1 allowed us to assess whether such belief confidence was related to the learning process in a meaningful manner, this design was ill-suited to investigate how people chose between exploration and exploitation. This is primarily due to the high proportion of rating trials, in which participants observed outcomes from both arms of the bandit, which allowed them to form a good enough estimate of the value of each arm of the bandit and making exploration unnecessary. As a result, their choices should arguably mostly be exploitative. In Experiment 2, which was highly similar to Experiment 1, we therefore reversed the ratio of trial types with most trials (75%) being choice trials. However, to allow insight into the evolution of value estimation during learning, we also collected a smaller percentage (25%) of rating trials, but for the reason mentioned above in this new version we no longer let participants observe the outcomes of these trials. During rating trials, people now judged their value beliefs and belief confidence for both lotteries. Which bandit had to be rated first was randomly chosen by the computer and could not be predicted by the participants. With a considerably larger number of choice trails, we expected more exploration trials in this experiment, i.e. trials in which people would not just classify their confidence as “guessing” if asked, but instead knew that they had chosen the lower-value option. The confidence scale was thus extended to range from “low confidence” to “high confidence” for this experiment, as is common practice in perceptual decision-making studies where errors are more likely to be detected albeit usually due to a different reason (responses are speeded whereas here participants consciously chose the suboptimal decision option to gather information). If participants were highly confident that they had made the suboptimal choice, they would report that they had “low confidence” they chose the optimal choice alternative on our scale. We made sure participants understood how to use the scale by instructing them carefully and allowing them to practice using this scale before moving onto the main task. Participants were thoroughly debriefed after the experiment and invited to comment on the task and ask questions. Issues with the decision-confidence scale were not raised. Prior to Experiment 2, participants completed two practice blocks, familiarizing them first with choice trials (5 trials) and then with a combination of both choice and rating trials (12 trials).

All testing was administered using a 24-inch monitor (16:9 aspect ratio) using the MATLAB toolbox PTB3 (Kleiner *et al.* 2007). Responses were given with an ordinary computer mouse. Prior to the analyses reported in this study, we excluded on average 2.2% of choice trials from the analyses of Experiment 1. Those were trials in which participants responded too slowly (±3σ rule calculated separately for each participant to exclude fast lapses and trials during which participants were most likely distracted; min = 0.7%; max = 3.3%). The same held for 1.7% of choice trials in Experiment 2 (min = 0.2%; max = 2.9%). No rating trials were excluded.

### Data analyses

The first set of analyses for Experiment 1 aimed to assess whether our new paradigm accurately captured belief learning over time. A second set of analyses assessed whether the uncertainty inherent in those beliefs—our concept belief confidence—constituted a meaningful judgment. We therefore attempted to link belief confidence to the more traditionally used concept of decision confidence using a linear, hierarchical regression model to predict decision confidence. We included participants’ last rated value-belief and belief-confidence ratings of both the chosen and the unchosen option as fixed effects, as well as the interaction of these estimates. Moreover, three control variables were included as fixed effects. The first variable was the objective accuracy of a trial. Decision confidence is an agent’s subjectively perceived accuracy of being correct. The objective accuracy of a trial should thus positively predict confidence, with higher confidence for correct trials. The second control variable were log-transformed RTs, given the “time heuristic” that suggests that reaction times (RTs) and confidence are negatively correlated across trials (e.g. Audley 1960; Moreno-Bote 2010; Hanks *et al.* 2011; Kiani *et al.* 2014). The third and last control variable was the number of the current trial within the block. We predicted that people’s decision confidence would increase over the course of each block, reflecting increasingly better choices. In addition to these fixed effects, the data were modeled with a random intercept and random slopes for all regressors. This regression model was fit to data from choice trials only with the *R* package “lme4” in combination with the *R* package “lmerTest.” The latter implements the Satterthwaite approximation, a formula that allows calculation of pooled degrees of freedom from variances of several independent normal distributions (Satterthwaite 1946), which in turn allows calculation of *P*-value estimates (for more detail see Schaalje *et al.* 2002). All predictors were *z*-transformed prior to being entered into the model to obtain standardized regression coefficients.

The key goal of Experiment 2 was to test for uncertainty-driven exploration. Here, we define exploration as choice trials in which participants chose the option they might reasonably believe to be lower-valued. Since value ratings for the individual arms of the bandit were only collected during rating trials, we extrapolated the current value rating for choice trials using the measurement taken at the most recent rating trial. An exploration trial is then defined as a trial in which the participant choses the arm of the bandit they rated as yielding lower rewards, whereas an exploitation trial is defined as choosing the option rated to yield higher values. We then fitted a logistic, hierarchical regression model to the choice-trial data of Experiment 2 to test for uncertainty-driven exploration. The dependent, binary variable expressed whether or not on the current trial people chose the choice option that they had previously rated as having a lower-value compared to the other option—our new operationalization of exploration. We included participants’ last rated belief confidence of both the higher- and the lower-value choice option as fixed effects, as well as the interaction of these estimates. Moreover, the difference in value (DV) for the choice options (high minus low) was included as a fixed effect. In addition to these fixed effects, the data were modeled with a random intercept and random slopes for all regressors. This regression model was again fit to data from choice trials only, using the *R* package “lme4”. All predictors were *z*-transformed prior to being entered into the model to obtain standardized regression coefficients.

In Experiment 2, we furthermore attempted to link inter-individual differences in people’s ability to trace uncertainty to their metacognitive efficiency. We obtained metacognitive efficiency measures, *M*-ratio, by fitting second-order signal-detection theory models to participants’ decision confidence data (Maniscalco and Lau 2012; Fleming and Lau 2014) using the MATLAB package HMeta-d (Fleming 2017). This approach automatically adjusts for differences in first-order performance. We furthermore fitted hierarchical, linear regression models that predicted belief confidence from the variance and mean of past outcomes, the current trial within the block, as well as the arm of the bandit. The standardized beta weights of the influence of the variance of past outcomes on belief confidence were used as an indicator for the extent to which people were capable of tracking uncertainty.

For the sake of simplicity, for all regression approaches in this study we report coefficients from the model that fitted the data best. However, a comprehensive list of models of varying degrees of complexity (10, 5, or 6 models, respectively) and a formal model comparison based on Bayesian Information Criterion (BIC) scores is included the Supplementary Material, Fig. S1, S2 and S3.

## Results

### Experiment 1

#### Participants formed value beliefs over time

**Figure 2.**
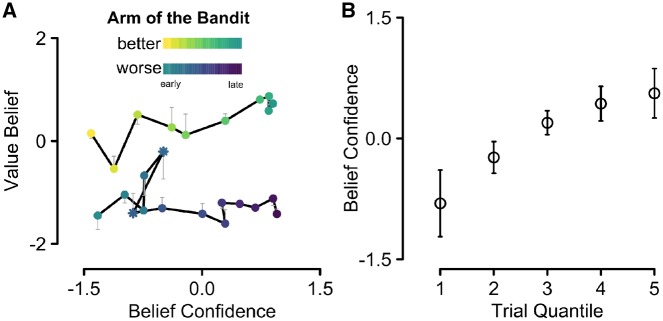
(**A**) Average traces of participants’ value belief and belief confidence ratings, given on the 2D grid scale for one example block. All ratings are *z*-transformed within-subject and then averaged across participants to reduce inter-individual differences in the use of the rating scales. The arm with the objectively higher rewards is shown in yellow to green (shown on 10 trials out of the block, corresponding to one data point each) and the other arm in blue to purple hues (shown on 14 trials out of the block, corresponding to one data point each). The brightness reflects the position of the data points within with block with brighter (yellow or blue) hues representing the earlier trials. The hairline arrows reflect the mean reward, calculated from the observed outcomes (objective mean value of the past outcomes). The length of the arrows is therefore proportional to the estimation error with longer arrows reflecting worse value estimates. (**B**) Belief confidence increased over blocks: the *x*-axis shows trial quintiles, calculated within each block ranging from the first (1) to the last (5) fifth of trials in each block. Belief confidence was *z*-transformed within-subject and then averaged across participants. The error bars reflect ±1 SEM.

In Experiment 1, we investigated how value belief and belief confidence rating evolved over time. We assumed that participants would adjust their value-belief estimates with each sample of evidence, presumably getting closer to the true value over time. At the same time, the level of confidence in these value estimates should increase in the course of learning. We found support for both of these predictions and address each in turn here. First, as expected we found that participants’ value estimates increased in accuracy over time. Value estimation accuracy was on average 11 points off during the first half of the blocks, and only 8 points off during the second half. This difference in estimation errors was reliable, *t*(20) = 9.7, *P *<* *0.001. This overall pattern of improvement can furthermore be seen in Fig. 2A, in which the gray hairline arrows are smaller for later trials in the example block (darker colors) compared to earlier trials (lighter colors). This figure furthermore shows that people perceived the arms of the bandit as more similar at the beginning of the block (the two traces are closer together) compared to the end of the block.

**Figure 3.**
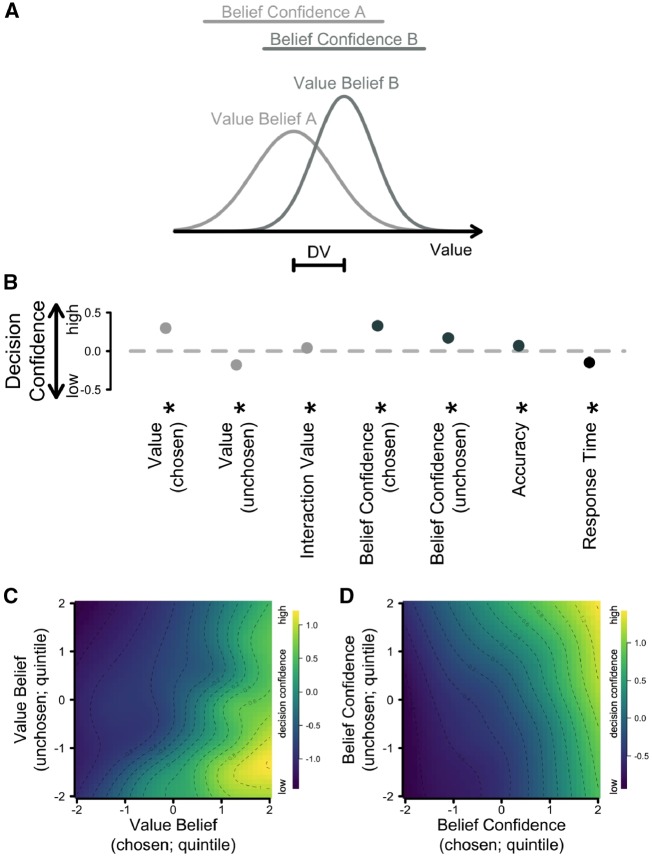
Hierarchical regression model used to predict decision confidence. (**A**) Schematic figure showing the noisy value representation for two objects. For the purpose of simplicity, each value belief is represented as a normal distribution with a mean (value belief) and a standard deviation (belief confidence). For these two overlapping choice options, Option B has a higher value than Option A, and also a more precise value representation (higher belief confidence). (**B**) Standardized, fixed regression coefficients from a hierarchical, linear regression model. Positive, higher parameter estimates reflect that an increment in this variable led to an increment in decision confidence. The error bars, which are almost entirely hidden behind the disks, reflect ±1 SEM. The light gray disks represent predictors linked to value, the dark gray disks represent predictors linked to belief confidence, and the black disks represent control variables. (**C** and **D**) Depict the influence of the key predictors on decision confidence for both value belief (**C**) and belief confidence (**D**). Lighter colors reflect higher levels of decision confidence. DV = difference in value.

Second, participants grew monotonically more confident in their value estimates (belief confidence) over the course of learning, *M*_early_ = 0.36 vs. *M*_late_ = 0.58, *t*(20) = 9.4, *P *<* *0.001 (see Fig. 2B). This increase in confidence is furthermore reflected in the traces in Fig. 2A, where later judgments (darker colors) lie closer toward the right end of the *x*-axis. However, the example traces also show that belief confidence could sometimes suddenly decrease over the course of a block, as reflected, for instance, in the two data points highlighted by asterisks in Fig. 2A: the darker colored asterisk lies further to the left compared to the lighter colored asterisk. Presumably, this happens whenever the newly sampled evidence leads to a larger update in value belief. Indeed, the larger the absolute difference between the currently observed outcome and the previously estimated value for the respective arm of the bandit, the larger the decrease in belief confidence, as reflected in individual correlations that were negative for 20 out of 21 participants, *r*s ≥ −0.46 and *r*s ≤ −0.01, and reliable for 16 out of 21 participants (*P*s < 0.01). Interestingly, such behavior goes against how a Bayesian agent would integrate information: every new piece of evidence, no matter how “far” it lies from the prior should lead to a reduction of the variance of the posterior (spread of the distributions in Fig. 3A). Our data therefore suggest that Bayesian integration might not be a suitable model for belief confidence. We explore this point in more detail in the Supplementary Materials, where a type of Bayesian filter (a particle filter) is directly compared to three reinforcement learning (RL) models to model people’s value learning and trial-by-trial updating of belief confidence.

Taken together, these findings suggest the paradigm used here is well suited to study the development of value beliefs and belief confidence over time: participants’ value estimates increased in accuracy and this was reflected in increases in belief confidence.

#### Linking belief confidence to decision confidence

Having established that our paradigm reflects meaningful changes in beliefs over time and uncertainty inherent in those beliefs, we then aimed to investigate the link between people’s (value) belief confidence—the key concept of interest in this study—and decision confidence. We have previously shown that decision confidence is a function of the DV (De Martino *et al.* 2013). The schematic diagram in Fig. 3A outlines this finding: two noisy value beliefs are represented as distributions, with their distance reflecting the DV. In the present study, we furthermore propose that belief confidence affects decision confidence. Belief confidence is reflected in the spreads of the two belief distributions; the narrower these distributions are, the more confident people judged their decisions. We therefore fitted a hierarchical, linear regression model to predict people’s decision confidence from their value-belief and belief-confidence ratings of both the chosen and unchosen arm of the bandit, the interaction of these estimates, and three control variables: the objective accuracy of a trial and log-transformed RTs. The standardized regression coefficients for the fixed effects are presented in Fig. 3B. We chose to present the best-fitting model here based on BIC scores (BIC = 5293.9). However, a detailed model comparison approach which included a range of models, both more parsimonious and more complex, is included in the Supplementary Materials. Some of these alternative models included the position of the trial within a block as a regressor (log-transformed).

As reported by De Martino *et al.* (2013), a larger DV was associated with higher decision confidence, as reflected in the significantly positive and negative regression weights for the value of the chosen, β = 0.30, *P *<* *0.001, and unchosen option, β = −0.18, *P *<* *0.001, respectively. These predictors furthermore showed a small but reliable interaction effect, β = 0.04, *P *<* *0.05. Figure 3C depicts the influence of both value-belief regressors on decision confidence as a 2D grid with lighter colors reflecting higher simulated decision confidence.

Critically, being confident in their value estimates also made people more confident in their decisions, both for the chosen, β = 0.33, *P *<* *0.001, and also the unchosen choice option, β = 0.17, *P *<* *0.001. The best-fitting model did not include an interaction between these two predictors. However, the prediction pattern formed by these two belief-confidence regressors is shown in Fig. 3D.

Moreover, two control variables were included in these regression models. First, the objective accuracy of the current trial was a binary variable that affected decision confidence positively, β = 0.07, *P *<* *0.001, as predicted. In other words, if participants picked the objectively higher-value option, they tended to be more confident in their choices. Second, the faster a decision, the higher people’s subjectively judged decision confidence, β = −0.15, *P *<* *0.001.

Taken together, the results of Experiment 1 suggest that our experimental setup allowed us to track the evolution of value beliefs over the course of learning and that belief confidence reflects meaningful insight into such learning. We moreover found that how certain people are in their value beliefs affected their decision confidence, in support of our first hypothesis.

### Experiment 2

Having established with the findings from Experiment 1 that belief confidence constitutes a meaningful measure of value uncertainty, and having linked belief confidence with decision confidence, we then addressed another key question of this study: would people use their belief confidence to arbitrate between exploration and exploitation? In Experiment 2, the proportions of choice (75%) and rating trials (25%) were reversed to allow for more exploration, whereas in Experiment 1, people often gained a sufficient amount of information from the lower-value bandit simply through observing outcomes during the rating trials, thereby lowering the need for active exploration of that decision option during choice trials. Indeed, this new design largely increased the number of trials in which the participants chose the lower-value option: participants on average chose the subjectively perceived lower-value option on 22.1% of all trials as opposed to only 13.3% of all trials for the previous experiment—supporting the view that they were exploring more.

#### Participants have meaningful insight into their gambling behavior

**Figure 4.**
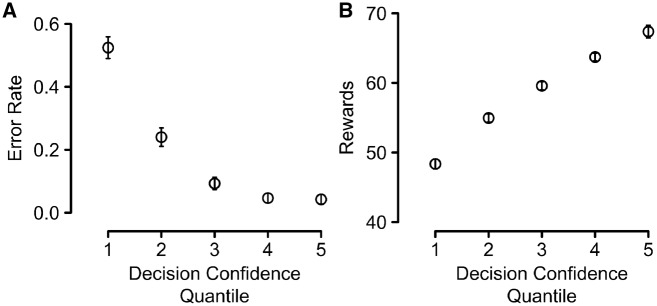
(**A**) Error rates and (**B**) average points won as a function of decision confidence. The data were binned according to decision confidence quintiles, which were formed within-subject. Errors are defined as trials on which people deviated from the ideal-observer model, i.e. trials on which they chose the arm of the bandit with so far the lower average in observed outcomes. All error bars are ±1 SEM for the respective *y*-axis values.

We first assessed whether decision confidence allowed any meaningful insight into people’s choice patterns, having now collected a considerably larger number of such choice trials. People made decisions on average 822 ms after the onset of the trial and chose the higher-value bandit—given previously observed outcomes—in on average 81.6% of all choice trials (this percentage is calculated based on objective value outcomes and not perceived value, as in the previous analysis). Overall, people showed good resolution in their decision-confidence judgments, i.e. their decision confidence varied with both the percentage of trials on which the higher-value option was chosen, as well as average reward points. Indeed, error rates (defined as the proportion of trials on which participants chose the lower-value option) differed reliably across confidence bins, *F*(1.7, 49.3) = 107.9, *P *<* *0.001, η*^2^_p_* = 0.79, with a reliable linear trend, *F*(1, 29) = 140.9, *P *<* *0.001, η*^2^_p_* = 0.83, see Fig. 4A. The same held for average rewards across decision-confidence quintiles, *F*(4, 116) = 117.0, *P *<* *0.001, η*^2^_p_* = 0.80, again with a reliable linear trend, *F*(1, 29) = 301.4, *P *<* *0.001, η*^2^_p_* = 0.91, see Fig. 4B.

#### Confidence-guided exploration

**Figure 5.**
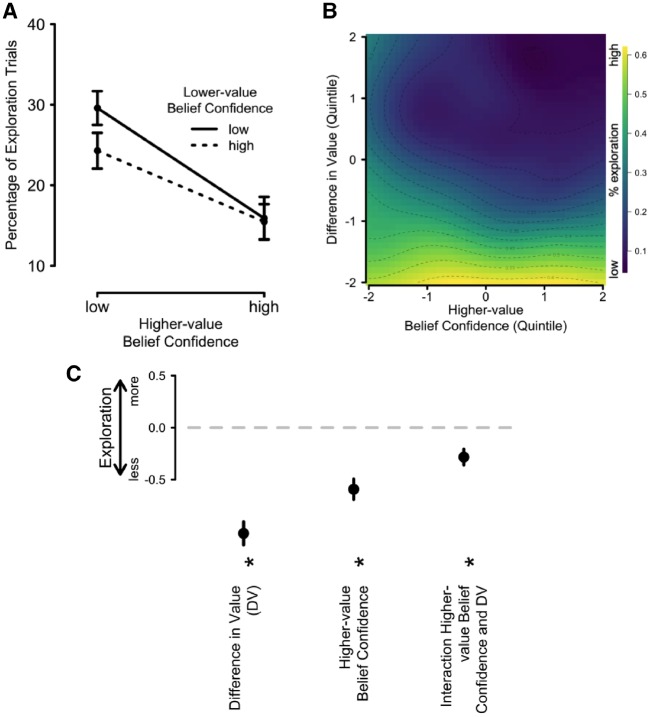
(**A**) Proportion of trials in which participants chose the lower-value option (exploration), as a function of the belief confidence of the higher- and lower-value options. (**B**) Proportion of trials in which participants chose the lower-value option (exploration trials), as a function of the belief confidence of the higher-value option and the DV. The dependent measure (exploration) is reflected in the color on the simulated grid, with lighter colors reflecting more exploration trials. (**C**) Standardized, fixed regression coefficients from a mixed-model logistic regression model, predicting exploration. Positive, stronger parameter estimates reflect that an increase in this variable led to a larger tendency to explore. All error bars reflect ±1 SEM. DV = difference in value.

To test whether participants arbitrate between exploration and exploitation based on their belief confidence, we fitted a hierarchical, logistic regression model to participants’ choice-trial data. The model predicts the probability of choosing the lower-value option (exploration) from the belief confidence of the higher-value option, as well as the unsigned DV as a control variable, which we defined as abs(*V_L_*_ _−_ _*V_R_*). This model was identified as the best-fitting model from a formal model comparison approach based on BIC scores, which we report in the Supplementary Materials along with several other models. The Supplementary Materials furthermore include the results from a model that also includes belief confidence of the lower-value option as a (non-significant) predictor as well as a (significant) interaction between the two belief confidences (see also Fig. 5A). However, this slightly more complex model did not fit the data as well (BIC = 11 114.2) as the model presented here (BIC = 10 923.9).

Critically, belief confidence of the higher-value option significantly predicted exploration, β = −0.59, *P *<* *0.001. This effect was negative, expressing that participants tended to explore more if their belief confidence was low, consistent with our prediction. The unsigned DV furthermore modulated choice significantly, β = −1.02, *P *<* *0.001, and negatively: the larger the absolute DV, the less participants chose to explore the lower-value option as arguably the overlap of the two value representations was small. Figure 5B presents how the DV and belief confidence of the higher-value choice option relate to exploration, with brighter colors reflecting higher proportions of exploratory choices. DV and confidence in the higher-value option did also interact reliably, β = −0.28, *P *<* *0.001. The standardized, fixed-effect regression coefficients are furthermore presented in Fig. 5C. Together, these findings suggest that if the value representation of the preferred option is noisier, people tend to explore the inferior choice option more, compared to when it is precise. We thus conclude that belief uncertainty—as measured through belief confidence judgments—guides the trade-off between exploration and exploitation.

#### Inter-individual differences in participants’ capability to track uncertainty

The increase in choice trials allowed us to obtain reliable measures of people’s metacognitive efficiency—the accuracy of their decision confidence judgments. The final question, which we aimed to address with our study was whether the degree by which people can accurately harness the level of uncertainty in their beliefs through confidence relates to their metacognitive efficiency, thus explaining some of the inter-individual variability reported in the literature.

To estimate the former, we first fitted a hierarchical, linear regression model simultaneously to all participants’ rating-trial data to assess the degree to which belief-confidence estimates were driven by the variability of the past, observed outcomes or other control variables [mean of the past outcomes, the current trial within the block (log-transformed), and the arm of the bandit]. Included in this model were two-way interactions between all variables except for the arm of the bandit, as well as the respective three-way interaction. A formal model comparison based on the BIC score (BIC = 17 231.2), this model fitted the data best. A range of other models are presented in the Supplementary Materials.

**Figure 6.**
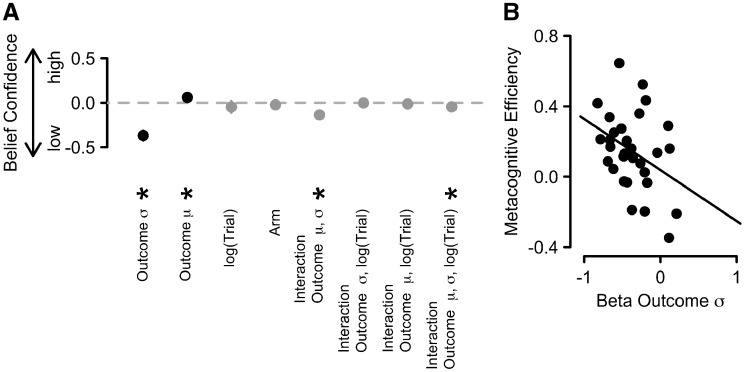
(**A**) Standardized, fixed regression coefficients from a hierarchical, linear regression model. Positive parameter estimates reflect that an increment in this variable led to an increment in belief confidence. The error bars, which are almost entirely hidden behind the disks, reflect ±1 SEM. The black disks represent predictors linked to the observed outcomes and the gray disks represent control variables and interaction effects. (**B**) Regression weights for the variance of past outcomes (ideal-observer model confidence) for each participant plotted against their metacognitive efficiency. σ = standard deviation; μ = mean.

The standardized regression weights for the fixed effects of all predictors are presented in Fig. 6A. This simple, normative model of how beliefs should be formed over time constitutes an ideal-observer model that can then be used to predict empirical responses. The influence of outcome variance on belief confidence was negative and reliably different from zero, β_sig_ = −0.37, *P *<* *0.001. The more variable past outcomes were, the less confident people became, consistent with our simple ideal-observer model. The mean of past outcomes had a weak positive, but also reliable influence on belief confidence, β_mu_ = 0.06, *P *=* *0.04, the higher the outcomes people had observed for this arm, the more confident they judged their beliefs. The other two control variables, the current trial within the block (log-transformed), β_logtrial_ = −0.05, *P *=* *0.46, and the arm of the bandit, β_trial_ = −0.02, *P *=* *0.24, did not reliably predict belief confidence. Out of all two-way interactions, only the interaction between the standard deviation and mean of all previously observed outcomes was reliable, β_sig__ __× __mu_ = −0.14, *P *<* *0.001, as was the three-way interaction between standard deviation and mean of all previously observed outcomes and the current trial, β_sig__ × __mu__ × __logtrial_ = −0.04, *P *<* *0.01. None of the other interactions were reliable, abs(βs)_ _<_ _0.014, *P*s >_ _0.54. Taken together, these findings suggest that people updated belief confidence similar to the simple ideal-observer model.

We then correlated the individual beta weights for the influence of outcome variance, β_sig_, with people’s metacognitive efficiency, log(*M*-ratio). We found that participants who were driven more in their belief-confidence estimates by the normative, ideal-observer belief confidence (outcome variance; i.e. larger negative regression weight for beta outcome σ) were more metacognitively efficient (higher metacognitive efficiency score), *r* = −0.37, *P *<* *0.05. In other words, people who were better at tracing the uncertainty present in the environment were better at distinguishing their own “good” and “bad” decisions, further linking the concepts of belief confidence and decision confidence. There was no such relationship between the beta weights for the influence of outcome mean, β_mu_, and metacognitive efficiency, *r *=* *0.02, *P *=* *0.94.

The results of Experiment 2 suggest that people use their belief confidence to arbitrate between two extreme modes of behavior, exploration and exploitation. We moreover found that people whose belief confidence matched that of an ideal observer closer also showed better insight into their own decisions, thus suggesting a link between uncertainty in the belief representations (belief confidence) and decision confidence, further shedding light on the internal signals and cues that give rise to confidence in a value-based decision-making context.

## Discussion

The present study used a novel variant of a bandit paradigm that allowed tracking of belief formation over time. We found that noisy value representations—especially regarding the higher-value option—led participants to explore more. Such uncertainty-guided exploration matches and further extends previous findings of uncertainty-driven exploitation (e.g. Frank *et al.* 2009; Badre *et al.* 2012). The key difference, however, was that we have demonstrated that it is possible to directly measure the uncertainty that serves as a cue to action selection using confidence judgments. Previous studies, on the other hand, have focused entirely on computational estimates of uncertainty. Our findings are further strengthened by our explicit measure of exploration. In previous studies, exploitation has commonly been operationalized as the act of choosing the same option compared to the previous trial—whether this is referring to discrete choice options (e.g., Kolling *et al.* 2012) or different decision strategies that are combined continuously (e.g. Frank *et al.* 2009). However, such an operationalization is unable to capture whether the agent chooses the option that he perceives to currently yield the best outcomes (exploitation) or not (exploration). Here, we defined as “exploration” a situation in which people choose an option they explicitly rated as inferior.

Confidence-guided exploration takes its place alongside a range of other findings that suggest that confidence forms a cornerstone of cognitive control (for reviews see Nelson and Narens 1990; Fernandez-Duque *et al.* 2000; Yeung and Summerfield 2012, 2014; Shea *et al.* 2014). For instance, research on metacognition in memory suggests that people use their internal, metacognitive signals to optimize learning, focusing on items they feel least confident about (Nelson and Leonesio 1988), even when these metacognitive judgments were objectively wrong (Metcalfe and Finn 2008; Hanczakowski *et al.* 2014). Similarly, findings from the error-monitoring literature suggest that people commonly slow down after committing an error (Rabbitt 1966), suggesting that they adopt a more accuracy-focused response regime to avoid further mistakes (Dutilh *et al.* 2012). Relatedly, confidence has been shown to serve as an internal teaching signal to support learning (Guggenmos *et al.* 2016) and metacognition has been proposed as a mechanism to guide people’s decisions to cognitively offload by setting reminders to avoid memory failures (Risko and Gilbert 2016). The notion that representations of uncertainty can be used by the brain to optimize behavior therefore extends and augments previous findings and discussions on metacognition.

We found that belief confidence predicted exploration in a linear way: the more uncertain people judged their value beliefs, the more likely they were to explore respective choice options. This finding is seemingly at odds with studies and theories that suggest confidence is related to curiosity in an inverse u-form shape (Kang *et al.* 2009; for a review of these findings see Butlin 2010). However, it should be noted that these studies used a full confidence scale, i.e. a scale that reaches from 0% confidence (certainly wrong) over 50% confidence (guessing) to 100% confidence (certainly correct). In our study, belief confidence ratings were given on a scale ranging from 50% to 100% confidence. We chose this scale because we collected value belief and belief confidence ratings concurrently and therefore did not expect any error detection. These findings can thus be reconciled considering only the curiosity ratings for the upper half of the scale used in Kang *et al.* (2009).

Our study furthermore linked the concept of (value) belief confidence, to the more traditionally studied concept of decision confidence. We found that participants with more accurate insight into their decisions tended to be driven more by the variability in their experienced outcomes in their belief confidence. Relatedly, we found that belief confidence had an effect on how some people judged their choices. Critically, this was found not only for the chosen option (being certain regarding the higher value of the option participants selected increased their decision confidence) but also for the belief confidence of the unchosen option: being certain about the value of the decision alternative people chose to forgo also increased their decision confidence. This finding fits and extends previous studies that reported that humans are capable of tracking counterfactual choice options (Boorman *et al.* 2011; see also Domenech and Koechlin 2015).

The finding that the belief confidence of both choice options affects decision confidence furthermore extends and links to previous results from our lab. In an initial study, we found that confidence in a value-based binary decisions (i.e. decision confidence in the current framework) was associated with activity in both the ventro-medial and the rostro-lateral prefrontal cortex (vmPFC and rlPFC) (De Martino *et al.* 2013) in which the former tracked both DV estimates and confidence and the latter only confidence. In a subsequent study (De Martino *et al.* 2017), instead of using binary choice we elicited confidence into value estimates (i.e. belief confidence according to the taxonomy used here) we found again an involvement of vmPFC (expanding into dorso-medial prefrontal cortex, dmPFC, according to a functional gradient) but no confidence signal in rlPFC. In light of the results presented here, we are tempted to suggest a possible dissociation of roles these two regions might play in the readout of uncertainty. In the choice study (De Martino *et al.* 2013), participants repeatedly chose between different snack items presented on screen, reporting their decision confidence with every choice, whereas value beliefs and belief confidence were not measured. Given the tight link between belief confidence in the chosen item and decision confidence that we showed in the present study, it is possible that the vmPFC activations that were observed in the binary choice study (De Martino *et al.* 2013) are mainly reflections of belief confidence (i.e. the uncertainty into the value estimation). This possibility is consistent with a subsequent finding in which belief confidence was directly measured in a functional magnetic resonance imaging (fMRI) study and in which activity in vmPFC was identified (De Martino *et al.* 2017; but see also Lebreton *et al.* 2015). An intriguing possibility is that rlPFC is involved only in decision confidence (or the subsequent use of such for the purpose of cognitive control; cf. Badre *et al.* 2012), i.e. when participants are requested to explicitly report the probability of an action to be correct. This matches the notion that the rlPFC is involved in the readout of metacognitive reports (usually measured in choice and not in estimation tasks), and it is supported by findings that show that coupling strength of the rlPFC with vmPFC is predictive of how efficiently uncertainty is read out for the metacognitive report (De Martino *et al.* 2013), as well as that gray matter volume in this area correlates with participants’ metacognitive accuracy (Fleming *et al.* 2010; for a review of the role of rlPFC in metacognition also see Fleming and Dolan 2012). This would suggest that low-level representations of uncertainty (measured by belief confidence here) are inherent to the computation performed by the cortical regions such as vmPFC for value beliefs (De Martino *et al.* 2013, 2017) or visual cortex for perceptual estimation (Fleming *et al.* 2012). However, following a choice, the uncertainty inherent in these low-level representations, together with uncertainty arising during the decision process such as response speed (Kiani *et al.* 2014) or familiarity and fluency (Koriat 1997) can in turn inform metacognitive reports that are instantiated in rlPFC.

In the present study, we proposed that belief confidence arises from the belief updating process, both of which we measured using explicit ratings. Belief confidence is likely to reflect both the stochasticity inherent in the lotteries (risk), as well as epistemic uncertainty, which decreases gradually through learning. Relatedly, a recent study by Meyniel *et al.* (2015a) proposed a learning paradigm that allows studying the development of confidence over time: participants were presented with a sequence of one of two possible stimuli. From time to time, participants had to estimate the probability that they were currently in a hidden state, which generated these stimuli with different probabilities and their confidence regarding the correctness of their probability judgment. Meyniel *et al.* (2015a) found that people were able to trace the evolving transition probabilities and that their confidence judgments reflected the uncertainty inherent in such a learning process, stemming from both the inherent stochasticity of the task, insufficient exposure, as well as the sudden transitions of states that happened throughout the experiment, bearing some similarities to the concepts of expected and unexpected uncertainty (e.g. Yu and Dayan 2005).

Furthermore, Bang and Fleming (2018) recently published a relevant fMRI study on the link between evidence certainty and decision confidence, that was similarly inspired by the conceptual proposal of Pouget *et al.* (2016). The authors used a random dot motion task and manipulated dot coherence to affect the reliability of the sensory evidence. Importantly, they found that certainty was encoded in posterior parietal regions, whereas decision confidence was encoded in medial prefrontal regions. The finding that different brain regions reflect these different representations of uncertainty are further support for the notion that the brain represents a multifaceted confidence system, the architecture of which we are only just beginning to grasp. The work presented here provides a suitable setup to study these two quantities in the context of value-based learning and furthermore demonstrates how these quantities influence learning. Future research might be able to capitalize on our paradigm to investigate whether belief confidence and decision confidence in the context of value-based decision making show a similar double dissociation at the neural level.

To conclude, our results provide a novel account of how uncertainty in value-belief judgments is constructed over time, and how people arbitrate between exploration and exploitation, with an uncertainty bonus biasing people toward exploration for the purpose of further information seeking (e.g. Daw *et al.* 2006; Frank *et al.* 2009). We suggest that our results carry implications for how the precision in the value representation for the chosen and unchosen option feeds into decision confidence, suggesting that uncertainty in the value-belief representations (belief uncertainty) also affects how confident people judge their choices, with higher decision confidence if decisions were based on precisely represented values of both the chosen and unchosen choice option. We showed that people who were better capable of tracing the uncertainty inherent in the environment also possessed higher metacognitive insight into their decisions. Our results therefore argue for a complementary role of decision and belief confidence judgments.

## Data Availability

The data and code are available for download under https://github.com/BDMLab/Boldt_et_al_2019.

## Supplementary Material

Supplementary Data
