# Supplementary material for: Confidence modulates exploration and exploitation in value-based learning
Source: Neurosci Conscious. 2019 May 8;2019(1):niz004. doi: 10.1093/nc/niz004 (PMC6505439; doi:10.1093/nc/niz004)
Supplement: Supplementary Data [file niz004_supp.docx]

**Supplementary Materials**

***Model Comparisons***

***Predicting Decision Confidence.*** To examine the effects of value beliefs and belief confidence on decision confidence, we compared several hierarchical regression models. A full description of these models can be found in Table S1, together with the resulting *BIC* scores in Figure S1. For the sake of simplicity, the main text presents only the best-fitting model (Model 8). Variables were included one by one and only kept in the model for the next step if they added value, that is if they reduced the *BIC*.

**Table S1:** Name and simplified formulas of the hierarchical regression models. Asterisks indicate interaction effects between variables. ε = error term.

| **Model** | **Formula** |
| --- | --- |
| Model 1 | DecConf ~ β_0_ + β_1_[Chosen Value] + ε |
| Model 2 | DecConf ~ β_0_ + β_1_[Chosen Value] + β_2_[Unchosen Value] + ε |
| Model 3 | DecConf ~ β_0_ + β_1_[Chosen Value] * β_2_[Unchosen Value] + ε |
| Model 4 | DecConf ~ β_0_ + β_1_[Chosen Value] * β_2_[Unchosen Value] + β_3_[Chosen Belief Confidence] + ε |
| Model 5 | DecConf ~ β_0_ + β_1_[Chosen Value] * β_2_[Unchosen Value] + β_3_[Chosen Belief Confidence] + β_4_[Unchosen Belief Confidence] + ε |
| Model 6 | DecConf ~ β_0_ + β_1_[Chosen Value] * β_2_[Unchosen Value] + β_3_[Chosen Belief Confidence] * β_4_[Unchosen Belief Confidence] + ε |
| Model 7 | DecConf ~ β_0_ + β_1_[Chosen Value] * β_2_[Unchosen Value] + β_3_[Chosen Belief Confidence] + β_4_[Unchosen Belief Confidence] + β_5_[Objective Accuracy] + ε |
| Model 8 | DecConf ~ β_0_ + β_1_[Chosen Value] * β_2_[Unchosen Value] + β_3_[Chosen Belief Confidence] + β_4_[Unchosen Belief Confidence] + β_5_[Objective Accuracy] + β_6_[log(RT)] + ε |
| Model 9 | DecConf ~ β_0_ + β_1_[Chosen Value] * β_2_[Unchosen Value] + β_3_[Chosen Belief Confidence] + β_4_[Unchosen Belief Confidence] + β_5_[Objective Accuracy] + β_6_[log(RT)] + β_7_[log(Trial Number)] + ε |
| Model 10 | DecConf ~ β_0_ + β_1_[Chosen Value] * β_2_[Unchosen Value] + β_3_[Chosen Belief Confidence] * β_4_[Unchosen Belief Confidence] + β_5_[Objective Accuracy] + β_6_[log(RT)] + β_7_[log(Trial Number)] + ε |

**Figure S1:** Resulting *BIC* values from model comparison approach from most parsimonious (Model 1) to most complex (Model 10). Model 8 (depicted in grey) was the best-fitting model and was reported in the main text for the sake of simplicity.
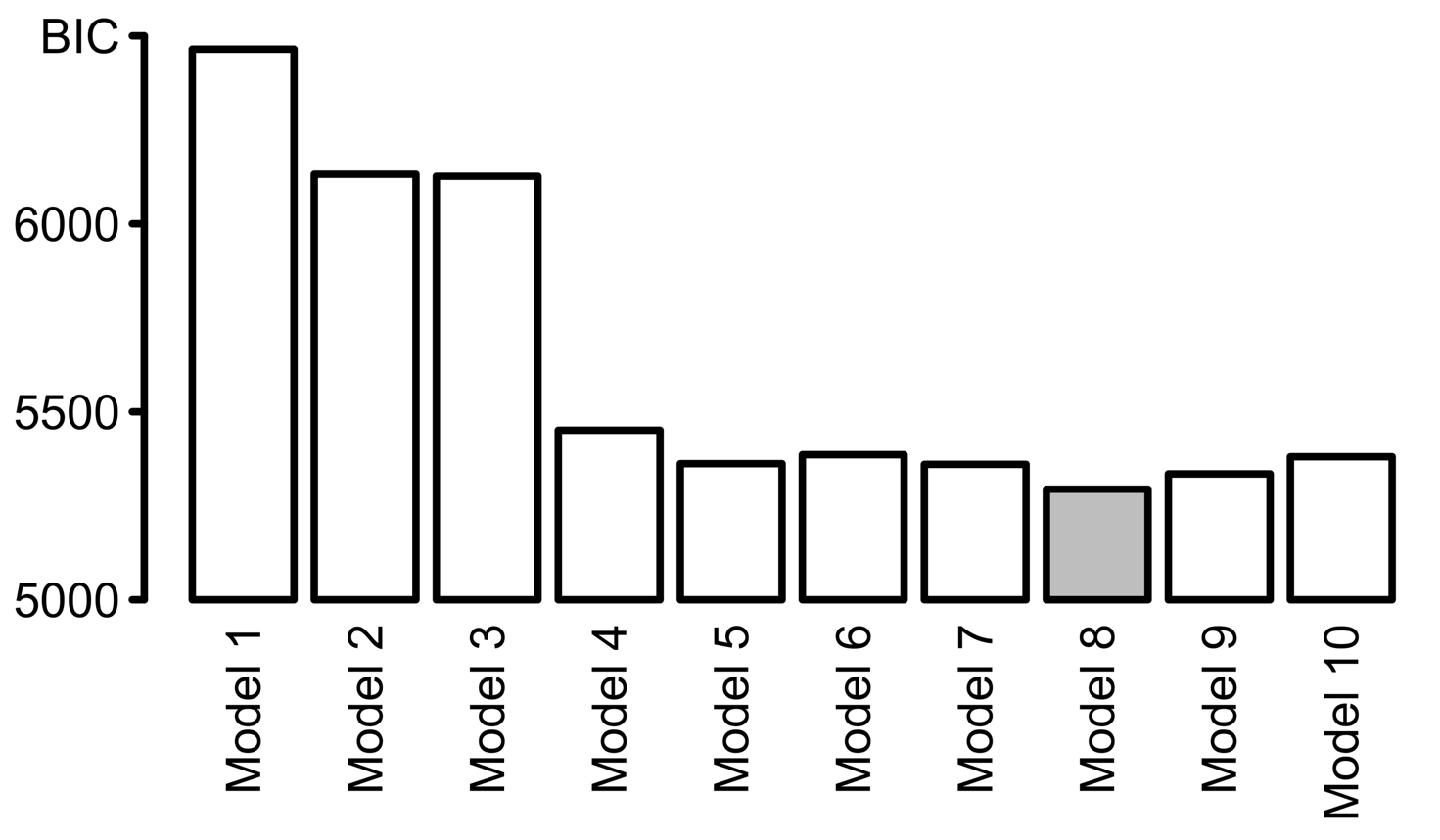


***Predicting Exploration.*** Several hierarchical regression models were conducted to examine the effects of belief confidence on exploration. The full description of these models can be found in Table S2, the resulting *BIC* scores are presented in Figure S2A. For this analysis, variables were again included one by one and only kept in the model for the next step if they added value, that is if they reduced the *BIC*. The best-fitting model identified was Model 3, also presented in the main text. Figure S2B furthermore shows the regression weights for the most complex model, Model 5. This model differed from the best-fitting Model 3 in that it also included the lower-value belief confidence as a predictor for exploration, as well as the interaction of this additional variable with the other two regressors in the model. Replicating the results reported in the main text, belief confidence of the higher-value option significantly predicted exploration, β = -0.54, *p* < 0.001. Again, this effect was found to be negative, reflecting that participants tended to explore more if their belief confidence was low. This relationship did not hold for the lower-value option, though, β = 0.08, *p* = 0.52. However, we found a reliable interaction between these factors, β = 0.21, *p* < 0.01, reflecting that the belief confidence associated with the lower-value option affected exploration only if the belief confidence associated with the higher-value option was low (see also Figure 5A in the main text). The unsigned difference in value furthermore modulated choice significantly, β = -1.02, *p* < 0.001, and negatively: The larger the absolute difference in value, the less participants chose to explore the lower-value option as arguably the overlap of the two value representations was small. Again, this finding replicated the results reported in the main text. DV and confidence in the higher-value option did also again interact reliably, β = -0.20, *p* = 0.04. None of the other interaction terms were reliable, abs(βs) < 0.09, *p*s > 0.20.

**Table S2:** Name and simplified formulas of the hierarchical regression models. Asterisks indicate interaction effects between variables. ε = error term.

| **Model** | **Formula** |
| --- | --- |
| Model 1 | DecConf ~ β_0_ + β_1_[Higher-value Belief Confidence] + ε |
| Model 2 | DecConf ~ β_0_ + β_1_[Higher-value Belief Confidence] + β_2_[Difference in Value] + ε |
| Model 3 | DecConf ~ β_0_ + β_1_[Higher-value Belief Confidence] * β_2_[Difference in Value] + ε |
| Model 4 | DecConf ~ β_0_ + β_1_[Higher-value Belief Confidence] * β_2_[Difference in Value] + β_3_[Lower-value Belief Confidence] + ε |
| Model 5 | DecConf ~ β_0_ + β_1_[Higher-value Belief Confidence] * β_2_[Difference in Value] * β_3_[Lower-value Belief Confidence] + ε |


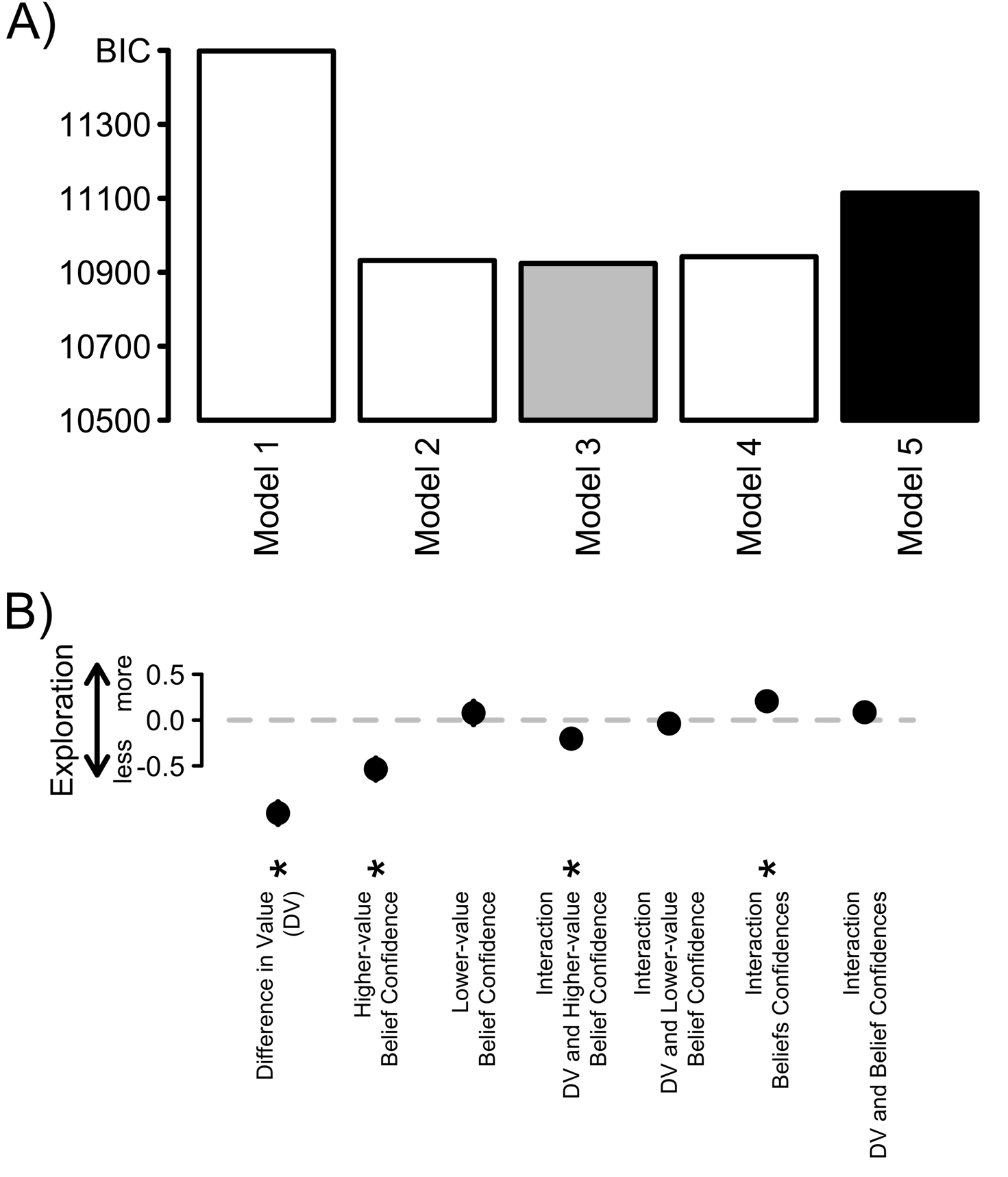


**Figure S2:** A) Resulting *BIC* values from model comparison approach from most parsimonious (Model 1) to most complex (Model 5). Model 3 (depicted in grey) was the best-fitting model and reported in the main text. The standardized, fixed regression coefficients resulting from Model 5 (depicted in black) are presented in B). Positive, stronger parameter estimates reflect that an increase in this variable led to a larger tendency to explore. DV = difference in value. All error bars reflect +/- 1 standard error of the mean.

***Predicting Belief Confidence.*** We compared several hierarchical regression models to assess the influence of the objective variance and mean of previously observed outcomes. Table S3 contains a full description of these models, together with their resulting *BIC* scores in Figure S3. Variables were included one by one and only kept in the model for the next step if they added value, that is if they reduced the *BIC*. The model from the main text, the best-fitting model in terms of *BIC*, is referred to as Model 6 here.

**Table S3:** Name and simplified formulas of the hierarchical regression models. Asterisks indicate interaction effects between variables. ε = error term.

| **Model** | **Formula** |
| --- | --- |
| Model 1 | BelConf ~ β_0_ + β_1_[Outcome Variance] + ε |
| Model 2 | BelConf ~ β_0_ + β_1_[Outcome Variance] + β_2_[Outcome Mean] + ε |
| Model 3 | BelConf ~ β_0_ + β_1_[Outcome Variance] * β_2_[Outcome Mean] + ε |
| Model 4 | BelConf ~ β_0_ + β_1_[Outcome Variance] * β_2_[Outcome Mean] + β_3_[log(Trial Number)] + ε |
| Model 5 | BelConf ~ β_0_ + β_1_[Outcome Variance] * β_2_[Outcome Mean] * β_3_[log(Trial Number)] + β_4_[Arm] + ε |
| Model 6 | BelConf ~ β_0_ + β_1_[Outcome Variance] * β_2_[Outcome Mean] * β_3_[log(Trial Number)] + β_4_[Arm] + ε |
| Model 7 | BelConf ~ β_0_ + β_1_[Outcome Variance] * β_2_[Outcome Mean] * β_3_[log(Trial Number)] * β_4_[Arm] + ε |

**Figure S3:** Resulting *BIC* values from model comparison approach from most parsimonious (Model 1) to most complex (Model 7). Model 6 (depicted in grey) was the best-fitting model and presented in the main text.
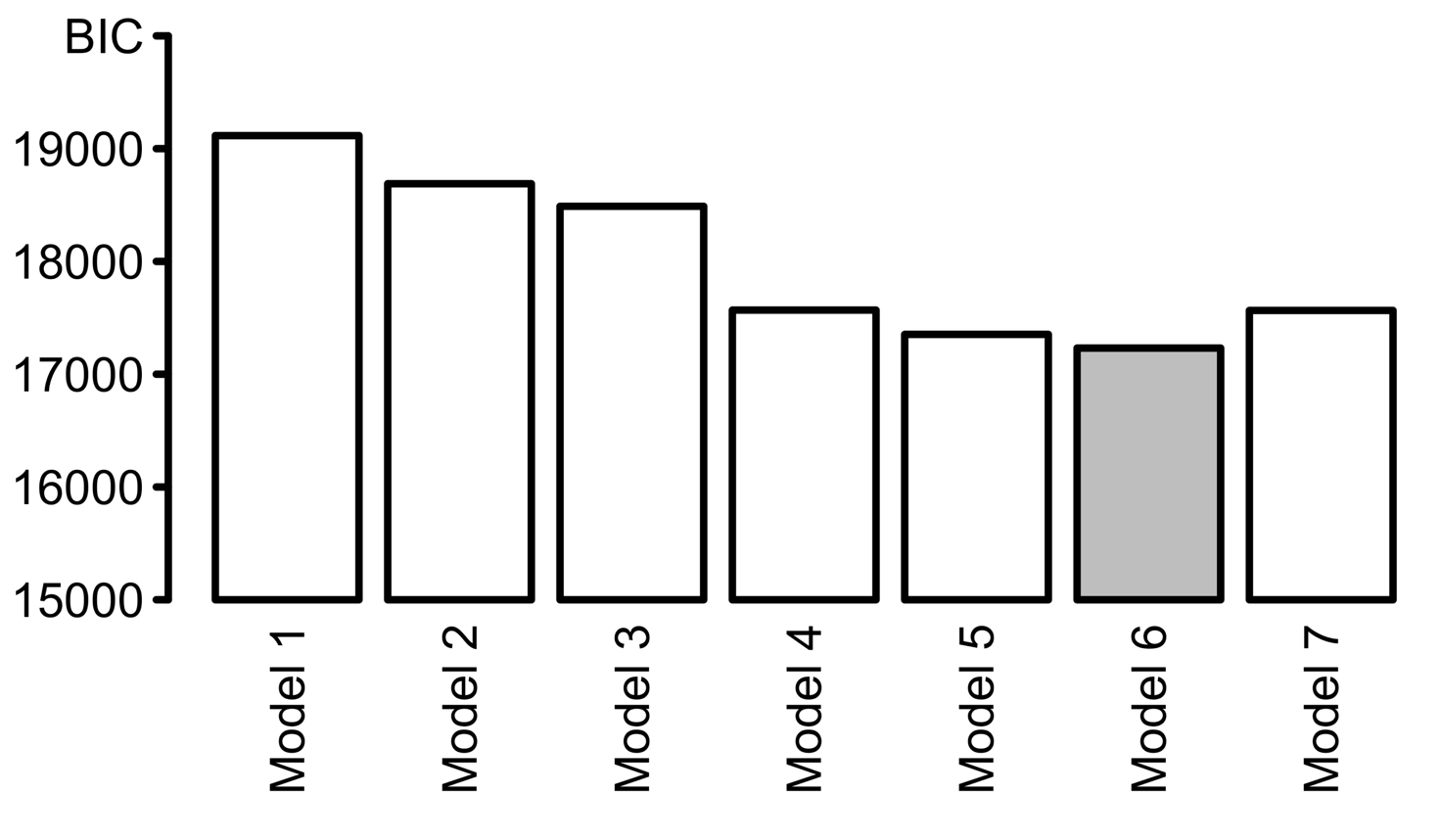


***Modelling Value-belief Learning***

In an exploratory analysis, we furthermore planned to draw on the rich literature on Bayesian modeling and reinforcement learning (RL) and compare different models of value-belief learning. We therefore conducted a direct model comparison for four computational models, drawing on the behavioral data collected in our study.

As a first model, we chose a particle filter, a Bayesian filter method related to the Kalman filter, but suitable for more complex, non-Gaussian data. This type of model is not only a flexible tool to study the behavior of complex, dynamic systems, but also allows for an organic implementation of value confidence as the width of the particle distribution, as detailed below. In addition, we chose three variants of an RL model in which the development of value belief over time was modeled using a delta rule. We augmented this model to account for value confidence in three different ways. A first (null) model assumed confidence to be randomly sampled from a gaussian distribution, a second model assumed increasing confidence over time and a third model assumed value-belief confidence to vary as a quadratic function of value belief (cmp. Lebreton, Abitbol, Daunizeau, & Pessiglione, 2015).

To foreshadow, for our particular dataset we found that an RL model which modelled value-belief confidence to increase steadily over time to fit the data best. We discuss to what extend this finding is reflecting a more general effect or whether it might have been influenced by task structure.

**Methods**

***Model 1: Particle Filter***

We used a Sequential Monte Carlo (SMC) approach, also know as a particle filter, to model the development of value and value-belief confidence over time. This approach has been developed to address the filtering problem, which describes a situation in which we try to infer the best estimate of the value of a system from potentially noisy outputs (e.g. Gordon et al., 1993). Particle filters extend Kalman filters for non-linear systems or systems with non-Gaussian noise. They are therefore the model of choice to deal with the continuous but bounded value space used in the current study. Particle filters have previously been used to describe human behavior (e.g. Sanborn, Griffiths, & Navarro, 2006; Daw, & Courville, 2008; Vul, Frank, Tenenbaum, & Alvarez, 2009).

At the beginning of each block, our model assumed *N* = 10,000 uniformly distributed particles across the value space that ranged from 0 to 100 points. This particle distribution can be regarded as the non-parametric posterior distribution and each individual particle can be regarded as a hypothesis about the true value of the respective arm of the bandit. Given that the bandits in our study had two arms (hidden states), each particle was two-dimensional. The particle distribution implicitly codes the two key aspects of the data: The value estimate is reflected in its central tendency, whereas value confidence is reflected in the width of the distribution.


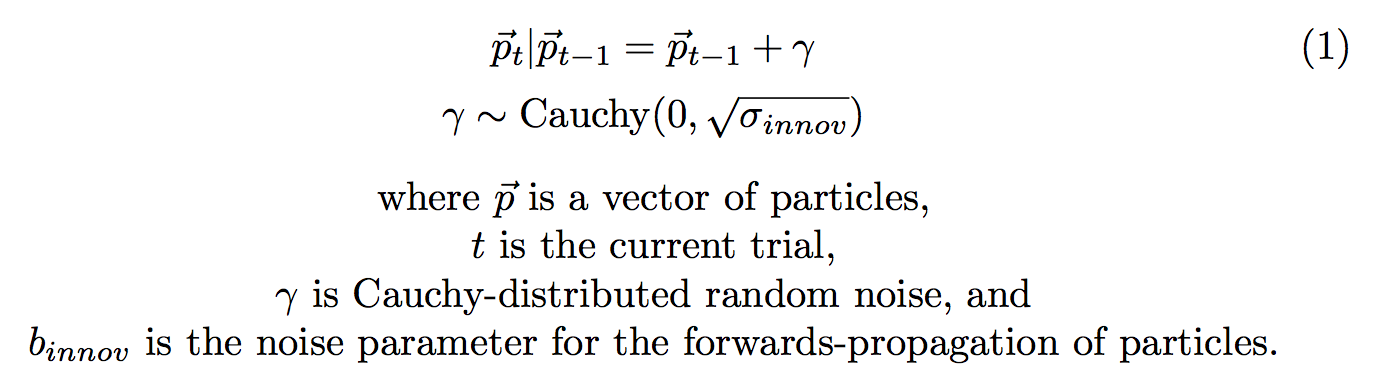
On each trial, particles are propagated forwards assuming a drift with Cauchy noise.


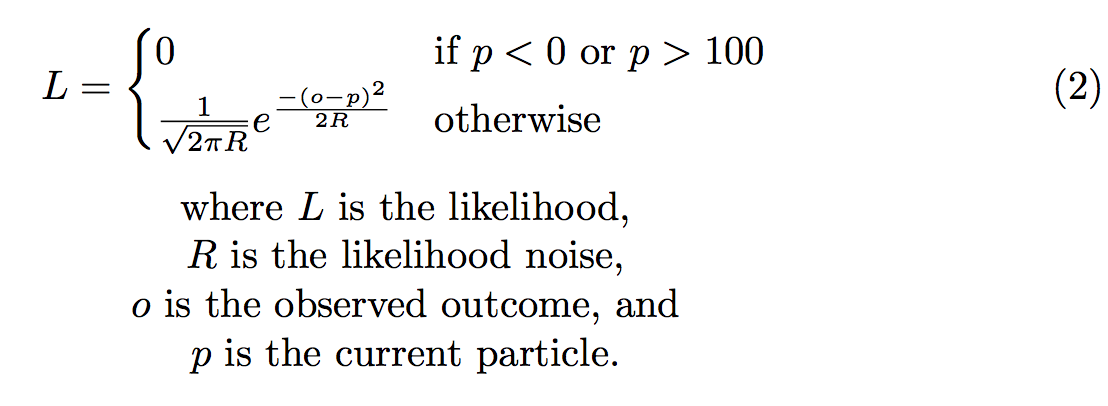
Whenever an outcome from the bandits is observed, each particle is evaluated against this outcome and a weight is assigned depending on the likelihood of the outcome under each particle hypothesis. The likelihood function used was a Gaussian distribution that was bounded between 0 and 100. In other words, because our value range only spanned these values, particles smaller or larger are possible due to the drift but are assigned a likelihood of 0.

The resulting likelihood values are taken as weights and particles for which the observed outcome was more likely (i.e. were closer in value space to the observed outcome) are assigned a higher weight. Crucially, the algorithm then resamples the particles according to their weight with replacement. Re-sampling is done for the currently-observed bandit, that is the one for which an observation was present on the given trial. The particle distribution now consists of *N* particles drawn across the value space. These particles are however no longer uniformly distributed but cluster around the currently most likely value estimate for the arm of the bandit. Ideally, this estimate gets sharpened over time. In our case, this step was repeated for the number of trials within the block.

It should be noted that in most machine-learning applications of SMC, it is commonly avoided to resample at every time point to avoid introducing too much variance into the computations. Instead, the algorithm resamples from time to time based on a function such as the effective sample size (ESS). However, trial-wise resampling is more biologically plausible as occasional resampling would cause rapid shrinking of the particle distribution and hence sudden decreases in belief confidence, which we have no reason to believe exists in our data.

Model 1 had four free parameters: The first free parameter was *b_ratio_*, which captured the relationship between the noise inherent in the initial particle distribution at time 0, *b_init_* (the second free parameter), and the noise parameter used for the forwards-propagation of particles, *b_innov_* (the scale parameter of the randomly distributed Cauchy variable):


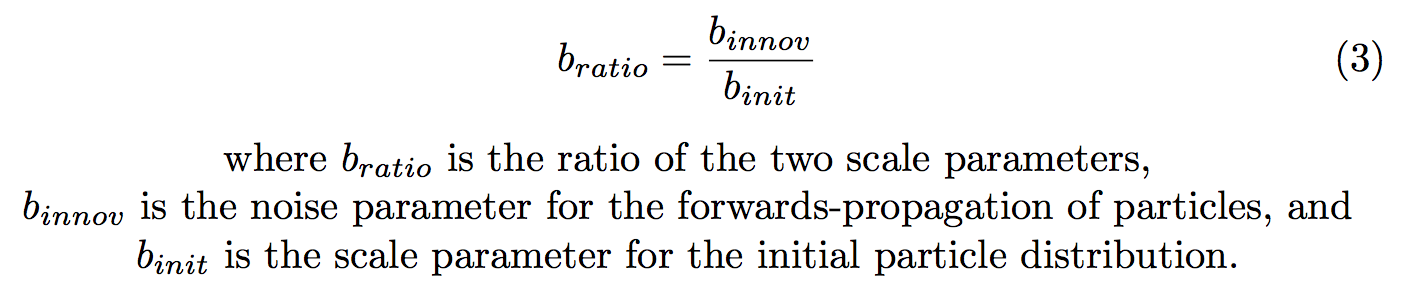


The third free parameter determined the width of the likelihood function from which the particle weights are calculated, *R*. The fourth free parameter, β, was a scaling parameter used in the cost function (see below) to fit the particle distribution to the empirical belief-confidence ratings. More specifically, β was multiplied with the empirical confidence to form a band around the simulated value.

***Models 2a, 2b, and 2c: RL models***

We furthermore fitted three reinforcement learning (RL) models to our value data. In all three models, learning was formalized using a delta rule:


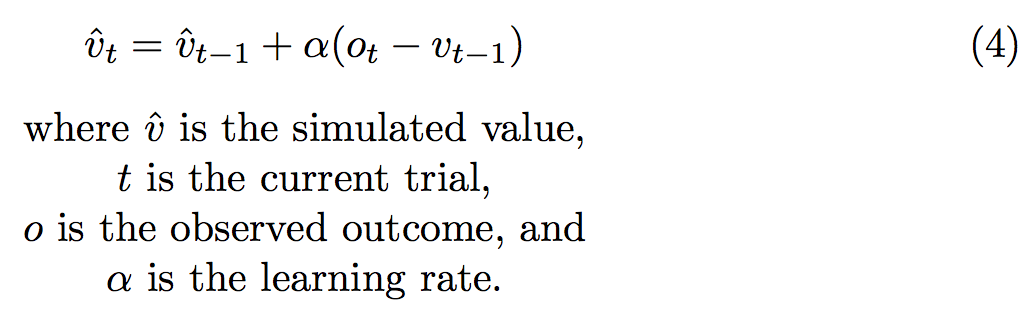


Moreover, each model implemented belief confidence differently. The first model served as a null model and assumed value confidence to be normally distributed across trials. To this end, value confidence was sampled from a Gaussian distribution with a mean, μ, and a variance, σ:


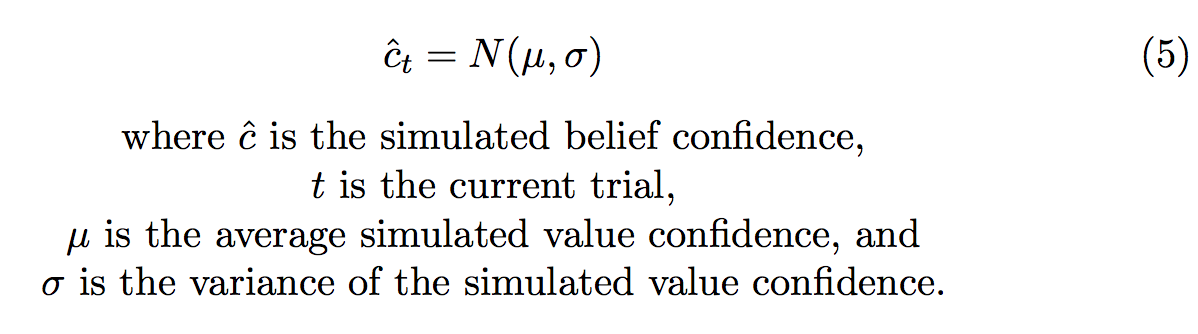


There were three free parameters: the learning rate, α, the mean, μ, and a variance, σ, of the belief-confidence distribution.

The second model assumed belief confidence to increase over time, inspired by our finding of increasing belief confidence across blocks reported in the main manuscript (see Figure 1B in the main text):


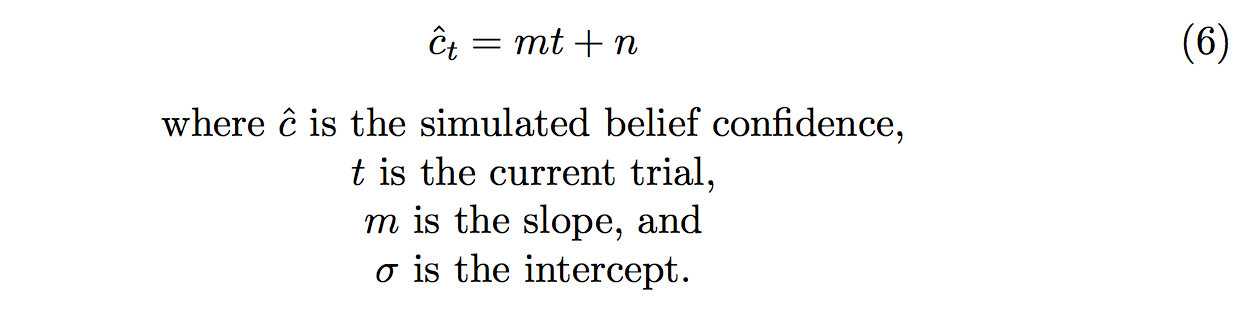


Again, three free parameters were fitted: the learning rate, α, the slope, *m*, and intercept, *n*, of the linear increase of belief confidence over time.

The third and final model assumed value confidence to vary quadratically with value, as has for instance been found by Lebreton and colleagues (2015). We calculated belief confidence based on the empirical value ratings.


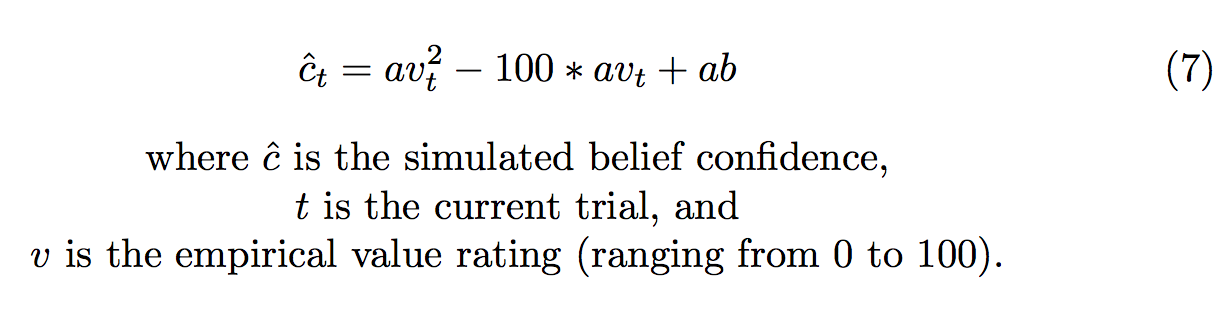


This model comprised only two free parameters: the learning rate, α, and the coefficient for the parabola, *q*.

***Parameter fitting and model comparison***

Due to the non-linearity of the particle filter extra caution has to be taken to not avid overfitting the noise. Approaches such as a simulated annealing algorithm are therefore not suitable and we instead resorted to a randomized search algorithm (RSA), using 35,000 random parameter combinations. We concentrated on the rating-trial data from Experiment 1, which allowed a better insight into value learning over time due to the larger proportion of rating trials (75%). We fitted the model to data from the same four blocks from all participants. The same approach was chosen for fitting the three versions of the RL model, for the sake of comparability. The to-be-minimized cost function for the particle filter was:


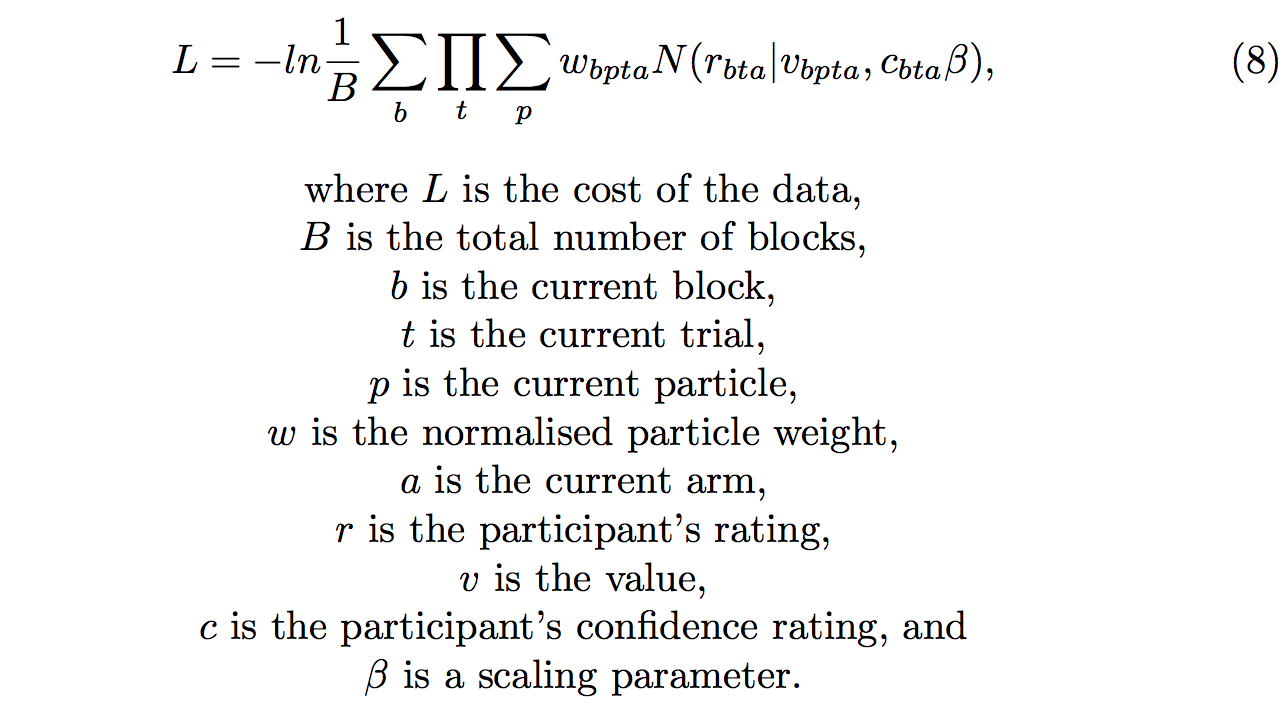


whereas the following cost function (without reference to the particle distribution) was used to fit the RL models to the data:


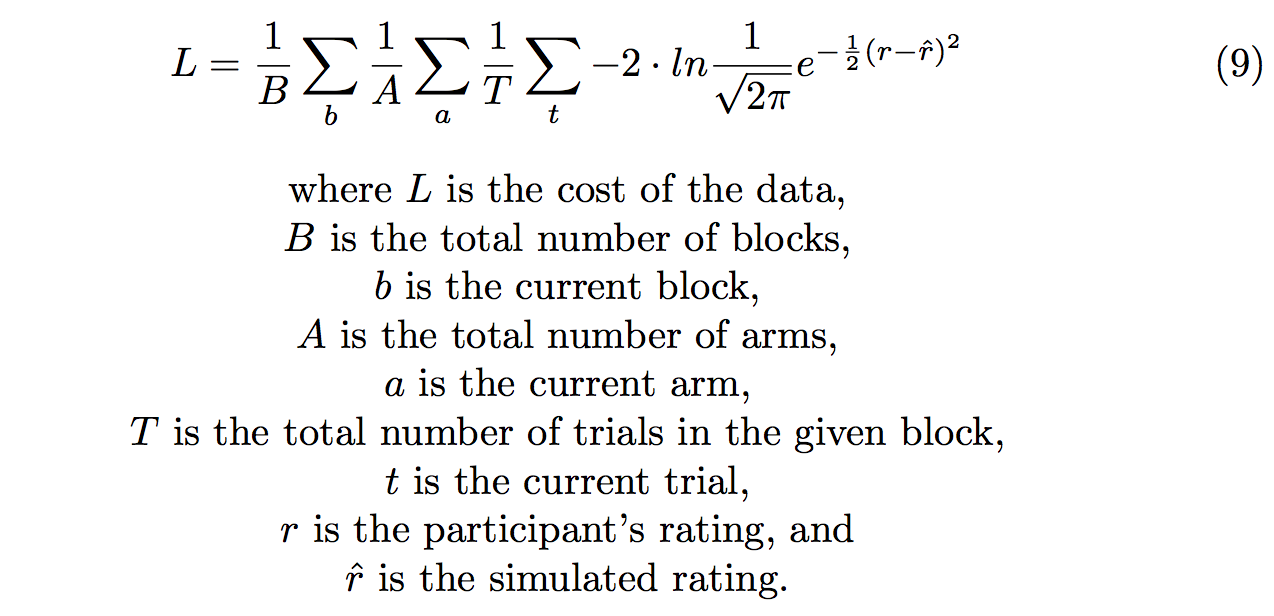


Belief confidence (both empirical and simulated) was multiplied with a factor 100 to place all data into the same space (ranging from 0 to 100) and to avoid under-fitting one aspect of the data.

A Bayesian Information Criterion (*BIC*) index was calculated based on the likelihoods and the model with the lowest *BIC* was deemed the best fitting one. In the sake of comparability, the likelihood was calculated in the same way for all models (see equation 9).

**Results**

We chose ranges for the parameters for the RSA based on initial piloting. All ranges together with the best-fitting parameters are presented in Table S4. Parameters were randomly sampled from a uniform distribution between those values.

Table S4: Ranges and best-fitting free model parameters

| **Model** | **Parameter** | **Minimum** | **Maximum** | **Best** |
| --- | --- | --- | --- | --- |
| 1: SMC | Noise Ratio *b_ratio_* | 0.00001 | 0.25 | 0.2466 |
|  | Initial Scale Parameter *b_init_* | 10 | 1700 | 812.4317 |
|  | Likelihood Noise *R* | 10 | 1700 | 361.8811 |
|  | Scaling parameter to capture belief confidence β | 20 | 900 | 25.6523 |
| 2a: Delta rule with value confidence sampled from a Gaussian | Learning rate α | 0 | 1 | 0.5300 |
|  | Mean of the value-confidence distribution μ | 0 | 1.2 | 0.7632 |
|  | Variance of the value-confidence distribution σ | 0 | 0.4 | 0.0113 |
| 2b: Delta rule with linearly increasing value confidence | Learning rate α | 0 | 1 | 0.5342 |
|  | Value-confidence slope *m* | 0 | 0.04 | 0.0246 |
|  | Value-confidence intercept *n* | 0 | 0.7 | 0.0908 |
| 2c: Delta rule with value confidence as a quadratic function of value rating | Learning rate α | 0 | 1 | 0.5387 |
|  | Coefficient for the parabola *q* | 0 | 0.002 | 0.0006 |

Figure S4 compares the empirical and simulated value and belief-confidence data for all four models for one example block. From visual inspection, it becomes obvious that all four models, especially the RL models, captured value learning well: The solid lines in Panels A) and B) lie close to the empirical ratings shown as red and blue xes. Seemingly, the SMC learned faster (i.e. the simulated value traces were more influenced by observed outcomes, shown as red and blue circles). It should be noted that the best-fitting learning rates for Models 2a-2c were very similar and resulted in visually indistinguishable plots, which is why Figure S4B only shows the results for Model 2a.


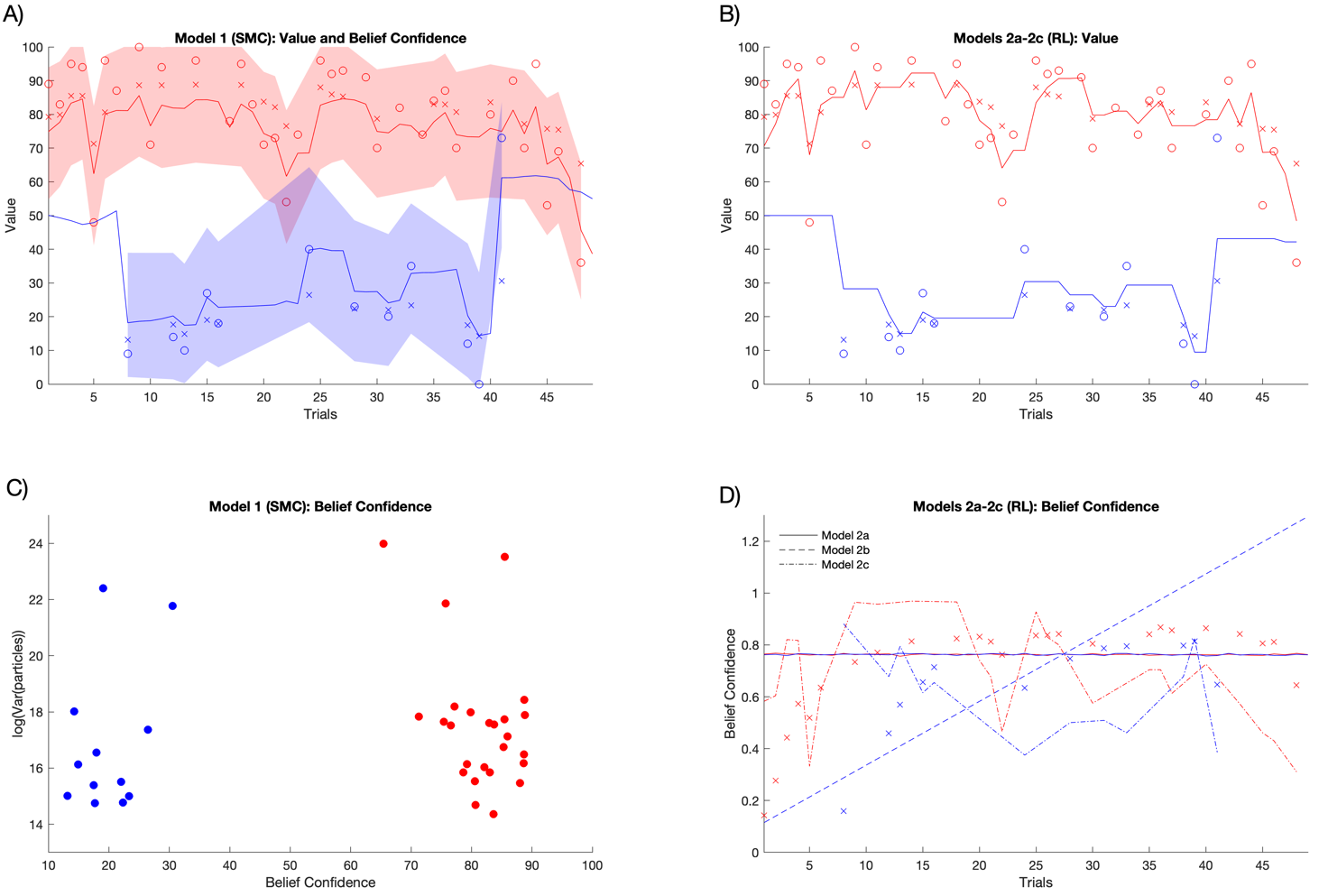
Figure S4: The same example block together with simulated data from the best-fitting parameter combinations for each of the four models. Red and blue reflect the different arms of the bandit. The solid lines represent the model’s value estimates over time. The circles represent observed outcomes. The xes show participants’ average ratings. The left panels depicts Model 1 (SMC). Panel A) The particle distributions are reflected in the partially overlapping, colored bands around the empirical ratings (showing the interquartile range, IQR, that is the difference between the 75^th^ and 25^th^ percentiles). The lines reflect the median of the particle distribution. Panel C) plots empirical belief confidence against the variance of the particle distribution, that is the simulated belief confidence. The latter measure was plotted as the logarithm to enhance visibility. The right panels (B and D) depict the value data and the belief-confidence data for Models 2a-2c (RL). In Panel B) only results for Model 2a are shown because the other models’ best-fitting results were visually indistinguishable.

Belief confidence, however, was not well captured by the SMC. Figure S4A clearly shows that the width of the particle distribution hardly varied over time. This is also reflected in Panel C), in which we plotted the empirical belief confidence against the variance of the particle distribution. To enhance visibility, this plot depicts the logarithm of the variance. A good model fit would have been reflected in a negative correlation but instead it is obvious that simulated belief confidence failed to capture the empirical pattern.

Simulated belief confidence generated by the RL models is presented in Figure S4D. Again, none of the three models fitted belief confidence particularly well in this example, however, it seems that Model 2b best captured the overall pattern of belief confidence with its upwards slope.

Figure S5 shows the *BIC* indices for the four models in comparison. Perhaps not surprisingly Model 2b, the RL model that assumed that confidence increased monotonically over trials, fitted the data best, followed by Model 2a, Model 2c, and lastly Model 1.


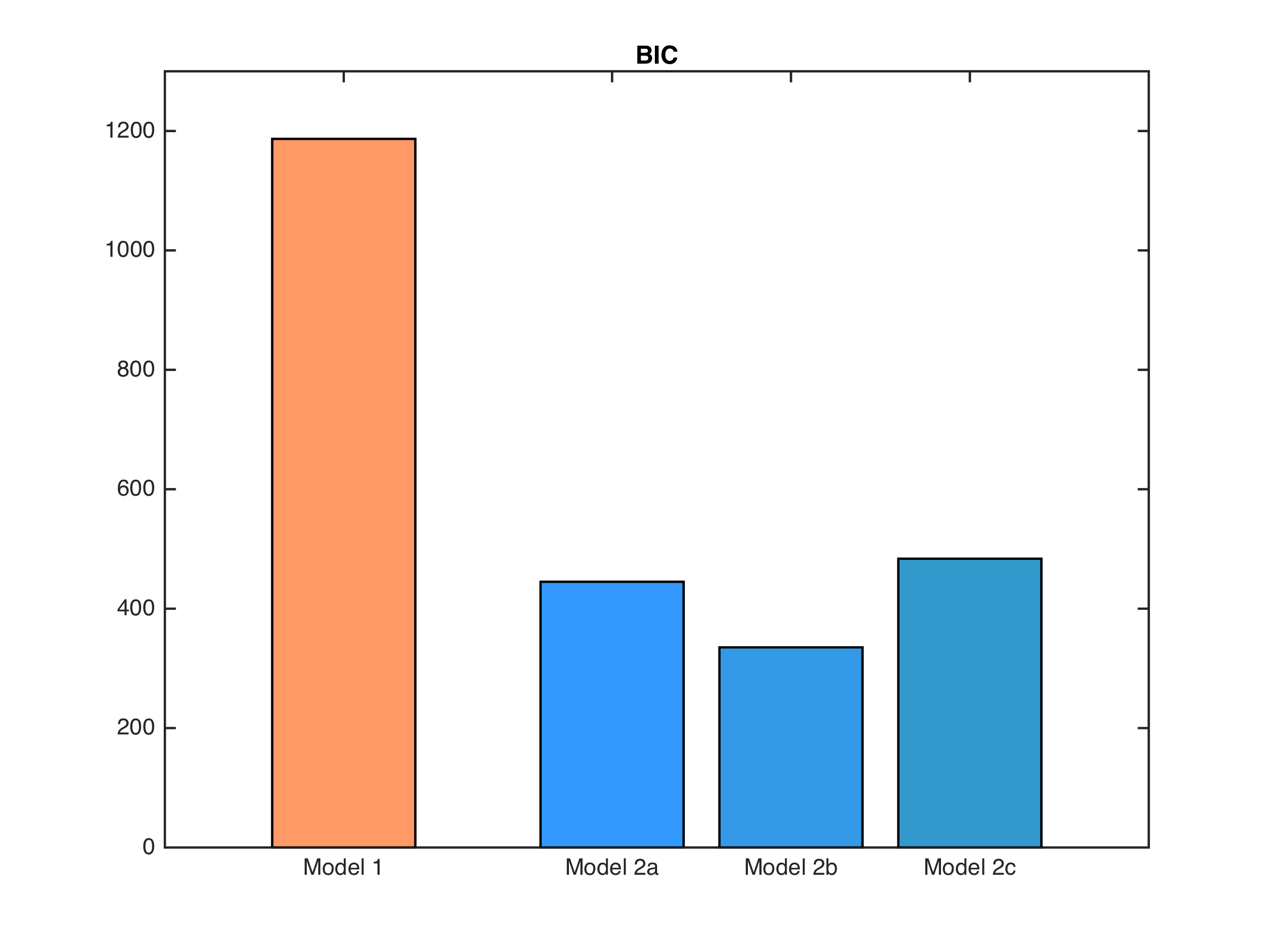
Figure S5: Bayesian Information Criterion (*BIC*) for the four computational models.

**Discussion**

Four models were directly compared to shed light on the evolution of value and belief confidence over time. Both the SMC model and the RL models accurately captured the development of value learning—the RL models slightly more successfully given that the SMC model seemed to learn slightly too fast.

However, the SMC model clearly struggled with fitting belief confidence. In this model, belief confidence was reflected in the width of the particle distribution, which is a nonparametric approximation of the posterior distribution. The simulated belief confidence is thus Bayesian confidence. But instead of following the empirical ratings, the algorithm produced belief confidence that varied mostly across the arms of the bandit. In the current study, we chose a particle filter instead of a Kalman filter not only because of our bounded, continuous value space, but also because it allowed us to implement a Cauchy rather than Gaussian drift. Our reasoning behind this was that in case of a Normal distribution, the step during which the particles drift apart reflects injection of uncertainty into the process. Critically, in case of normally distributed noise, the uncertainty is balanced with the information contained in the process. Rather than using a Gaussian distribution, we therefore chose a Cauchy, which has a ‘fat’ tail and should thus lead to larger updates. However, from our results it becomes apparent that the uncertainty in the particle distribution did not adequately in- and decrease over time. The main problems seem to stem from trials in which the observer faced surprising evidence, that is value outcomes that differ substantially from the currently-held value estimate. In the particle filter, such outliers are discounted when integrated into the posterior, however they still shrink the widths of the posterior independent of how unlikely they are. This behavior by the model is drastically different from how humans solve our task, who in our task decrease their belief confidence upon encountering outlying information. This basic finding suggests that Bayesian confidence might in general not be a good approximation for human belief confidence.

One of the RL models, on the other hand, produced the closest approximation of belief confidence (Model 2b). This model assumed that belief confidence would increase monotonically over time. However, from visual inspection of Figure S4D, it becomes apparent that there are many aspects of belief confidence that this model could not account for and that its fit to the data was only slightly better than the other contenders’ fits. This was probably due to the fact that in our design the bandits’ payoffs did not change over time and therefore confidence is only expected to increase over time. Future research using a different experimental paradigm designed to discriminate the predictions of different models and more extensive modeling work will be needed to address these questions.

**References**

Daw, N. D., & Courville, A. C. (2008). The pigeon as particle filter. *Advances in Neural Information Processing Systems*, 369–376.

Gordon, N. J., Salmond, D. J., & Smith, A. F. M. (1993). Novel approach to nonlinear/non-Gaussian Bayesian state estimation. *IEE Proceedings F Radar and Signal Processing*, *140*(2), 107. http://doi.org/10.1049/ip-f-2.1993.0015

Lebreton, M., Abitbol, R., Daunizeau, J., & Pessiglione, M. (2015). Automatic integration of confidence in the brain valuation signal. *Nature Neuroscience*, (July). https://doi.org/10.1038/nn.4064

Sanborn, A. N., Griffiths, T. L., & Navarro, D. J. (2006). A More Rational Model of Categorization. In *Proceedings of the 28th Annual Conference of the Cognitive Science Society* (pp. 1–6).

Vul, E., Frank, M. C., Tenenbaum, J. B., & Alvarez, G. (2009). Explaining human multiple object tracking as resource-constrained approximate inference in a dynamic probabilistic model. *Advances in Neural Information Processing Systems, 22*, 1–9.
